# Potato (*Solanum tuberosum* L.) can be grown safety on human consumption in slight Hg-contaminated soils across China mainland

**DOI:** 10.1038/s41598-020-65430-1

**Published:** 2020-05-20

**Authors:** Bo Yang, Yi Gao, Chunxue Zhang, Jiarui Han, Yige Liu, Xiangqun Zheng

**Affiliations:** 1 Agro-Environmental Protection Institute, Ministry of Agriculture and Rural Affairs, Tianjin, 300191 China; 2 Chinese Academy of Agricultural Sciences, Beijing, 100081 China; 3 College of Agriculture, South China Agricultural University, Guangzhou, Guangdong, 510642 China

**Keywords:** Environmental sciences, Environmental chemistry, Pollution remediation

## Abstract

Mercury (Hg) exposure poses serious health risks to humans, resulting in extensive investigations examining Hg accumulation, biotransformation and uptake in crops. In this investigation, Hg accumulation in potato tubers due to bioaccumulation processes was determined and bioconcentration factors affecting bioaccumulation were identified using a greenhouse experiment. Our results showed that the percentage of available Hg concentrations from total Hg in soil samples were less than 1.2%, indicating that soils used in our experiment exhibited a high binding strength for Hg, with alkaline soil recording the lowest available Hg/total Hg ratio. Results indicated that soil type and Hg treatment, as well as their interactions, significantly affected Hg accumulation in potato tubers (P < 0.01). Importantly, our results also indicated that potatoes grown in soil with a Hg concentration two times higher than the Chinese Environmental Quality Standard exhibited no obvious toxic effects on humans; Bioconcentration factors (BCF) values (<0.04) suggested that potatoes can be considered as a low Hg accumulating species and suitable for human consumption. Potato yields in acidic soil were lower than those in neutral or alkaline soils, making this medium unsuitable for growth.

## Introduction

Mercury (Hg) has been listed as one of the ‘ten leading chemicals of concern’ by the WHO^1^, and it is believed that more than 8 million people are exposed to Hg contamination globally^2^. Soil contaminated by Hg is a serious issue in Asia countries, with China being considered as the world’s largest producer and consumer of Hg^3^. A nationwide survey of Hg levels in soil in China recorded 1.6% of samples to contain Hg contamination^4^. High concentrations of Hg and its associated compounds in soil are highly toxic, due to its bioaccumulation, biological toxicity and long residence time in the environment^2,5^. Hence, there is an urgent need for soil remediation in order to reduce Hg risks.

Hg contamination and toxicity, and its transport into and from plants to higher organisms via the food chain is a serious area of concern^6,7^. The chronic consumption of low-dose Hg in humans can result in organ dysfunction, leading to systemic toxicity^8^. Research in China has shown that crops grown in contaminated soil, such as rice^9^, wheat^10^ and vegetables^11^, may contain a certain level of Hg. As root vegetables are directly exposed to Hg-contaminated soils, these crops have been recorded to have a greater level of Hg accumulation than other crops^12,13^. Due to the accumulation of Hg in agricultural products, it is imperative that the transfer of soil Hg into the food chain is reduced.

The root vegetable potato (*Solanum tuberosum L*.) contains high levels of starch, a wide variety of vitamins and has a low calorie content^14^. This vegetable, ranked as the fourth leading food crop in the world^15^, is widely distributed in China. Potato is commonly cultivated in four different agro-ecological regions of China: the Central plains (5%), the southern region (7%), the southwestern region (39%) and the northern region (49%)^16^. The recent guideline released by the Chinese Ministry of Agriculture proposed that potato consumption as a staple food is estimated to reach 30% of the overall potato intake by 2020^17^. As previously highlighted, efficiency of root Hg uptake is largely dependent on Hg bioavailability in soils^18^, and measurement of total Hg in a soil may not provide adequate data to assess potential soil toxicity^19^. Bioavailable Hg in soils to plants significantly varies with soil characteristics, cation exchange capacity, Fe and Al oxides, organic matter and pH^20^. The exchangeable fraction of Hg in a soil, representing fractions which are available and more mobile for crop uptake, is generally determined using single extractions^21^. Numerous extractants, such as water, chelating solutions, salt solutions and diluted acid solutions, have been adopted to examine available heavy metals in plants^22^. Among these, ethylenediamine tetraacetic acid (EDTA) is widely used as it can form a strong complex with almost all heavy metal ions^23,24^.

As China comprises broad geochemical landscapes and geologically diverse areas, a wide range of soils types (e.g. acidic red soil, calcareous soil and paddy soil) are distributed in different climatic zones. With different proportions of soil minerals, the mechanism of Hg enrichment and transformation can differ between soil types, resulting in different performances of bioavailable Hg in both soil and plants. As food is generally consumed within the local production area in China, the role soil type plays in Hg uptake by potato plants is important for different potato growing areas. In this study, we examined Hg uptake from different types of cultivated soils using pot experiments. The main aims of this study were: (i) to assess the transfer behaviour of Hg in potatoes from 18 different soil samples; (ii) to measure the content of bioavailable Hg in different soil types using the EDTA method; and (iii) to determine important bioconcentration factors in different soil types to guarantee the safe consumption of potatoes in China.

## Results

### Changes in the Hg bioavailability of soils

Comparison results for available and total Hg ratios in the different treatment groups before potato planting and after harvesting (Table 1) all recorded a decrease, except for the CK treatment. The maximum reduction value (0.74%) was recorded in Hebei soil, with LW_Hg_ treatment and ratio results being less than 1.5% before and after potato planting for all treatment groups. These results indicated that the majority of Hg in soils was displayed as a non-mobile fraction. Total and available Hg ratios all declined for the three soil types (acidic, neutral and alkaline), with neutral soils recording the greatest level of decline. Additionally, correlation analysis results indicated that available Hg and total Hg ratios recorded significant positive correlations (r = 0.894, p < 0.001), and total Hg was the important parameter affecting the availability of Hg in the tested soils (Table 2).

**Table 1:** Available and total Hg ratios in the soils before transplant and after harvest of potato.

pH	soil location	ratios (before planting)			ratios (after harvest)		
		CK (%)	LW_Hg_ (%)	HG_Hg_ (%)	CK (%)	LW_Hg_ (%)	HG_Hg_ (%)
<6.5	Guangdong	0.70 ± 0.07	0.55 ± 0.23	0.46 ± 0.03	1.02 ± 0.20	0.46 ± 0.06	0.58 ± 0.08
	Anhui	0.82 ± 0.25	0.48 ± 0.07	0.49 ± 0.19	1.11 ± 0.51	0.49 ± 0.19	0.28 ± 0.07
	Hubei	1.35 ± 0.21	0.56 ± 0.17	0.49 ± 0.08	0.68 ± 0.11	0.48 ± 0.09	0.31 ± 0.01
	Heilongjiang	0.47 ± 0.10	0.51 ± 0.03	0.52 ± 0.04	1.05 ± 0.28	0.43 ± 0.06	0.33 ± 0.05
	Hainan	0.91 ± 0.20	0.51 ± 0.11	0.38 ± 0.04	1.10 ± 0.36	0.50 ± 0.08	0.22 ± 0.06
	average	0.85 ± 0.16 aA	0.52 ± 0.12aB	0.47 ± 0.14aB	0.99 ± 0.30 aA	0.47 ± 0.09aB	0.34 ± 0.05aB
6.5-7.5	Hunan	0.79 ± 0.23	0.35 ± 0.14	0.26 ± 0.02	0.68 ± 0.57	0.31 ± 0.06	0.25 ± 0.05
	Zhejiang	0.86 ± 0.48	0.49 ± 0.07	0.25 ± 0.03	0.93 ± 0.59	0.25 ± 0.06	0.14 ± 0.03
	Yunan	0.81 ± 0.31	0.50 ± 0.10	0.24 ± 0.06	0.91 ± 0.27	0.38 ± 0.04	0.17 ± 0.04
	Jiangsu	0.92 ± 0.13	0.37 ± 0.02	0.26 ± 0.07	1.11 ± 0.08	0.29 ± 0.03	0.26 ± 0.03
	average	0.85 ± 0.30 aA	0.43 ± 0.33aB	0.25 ± 0.05bC	0.91 ± 0.38 aA	0.31 ± 0.05abB	0.20 ± 0.04bB
>7.5	Jilin	1.01 ± 0.48	0.13 ± 0.04	0.13 ± 0.03	1.23 ± 0.11	0.16 ± 0.04	0.11 ± 0.02
	Beijing	1.10 ± 0.09	0.19 ± 0.04	0.15 ± 0.04	1.28 ± 0.41	0.18 ± 0.05	0.11 ± 0.02
	Sichuan	0.44 ± 0.16	0.35 ± 0.04	0.18 ± 0.01	0.75 ± 0.40	0.25 ± 0.05	0.13 ± 0.02
	Xinjiang	1.08 ± 0.55	0.27 ± 0.10	0.20 ± 0.04	1.05 ± 0.31	0.22 ± 0.08	0.24 ± 0.07
	Hebei	1.05 ± 0.19	0.39 ± 0.05	0.14 ± 0.03	1.18 ± 0.45	0.10 ± 0.05	0.09 ± 0.05
	Shaanxi	0.52 ± 0.22	0.41 ± 0.02	0.16 ± 0.02	0.89 ± 0.27	0.30 ± 0.11	0.10 ± 0.01
	Henan	1.22 ± 0.24	0.29 ± 0.01	0.20 ± 0.02	1.44 ± 0.10	0.22 ± 0.08	0.11 ± 0.01
	Shanxi	1.05 ± 0.41	0.15 ± 0.02	0.16 ± 0.03	0.93 ± 0.59	0.27 ± 0.03	0.11 ± 0.05
	Ningxia	1.46 ± 0.60	0.14 ± 0.08	0.14 ± 0.02	1.37 ± 0.17	0.35 ± 0.12	0.14 ± 0.04
	average	0.99 ± 0.32 aA	0.26 ± 0.05bB	0.16 ± 0.03cB	1.13 ± 0.32 aA	0.23 ± 0.07bB	0.13 ± 0.07bB

*Mean ± SD, different small letters within the same column and different capital letters within the same row for each treatment indicate a significant difference at p < 0.05 by Student’s multiple range tests.

**Table 2:** Correlation coefficients between soil total Hg concentration, soil available Hg concentration and Hg concentrations in potato edible parts.

Hg content	edible-Hg	Total-Hg	Available-Hg
edible-Hg	1		
Total-Hg	0.553***	1	
Available-Hg	0.340	0.894***	1

***p < 0.001

### Total Hg content in potato

**Figure 1:**
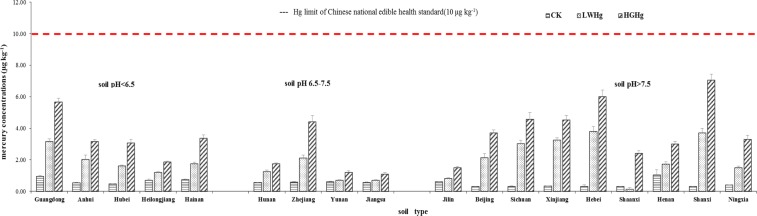
Hg content in the edible part of potato cultivars in 18 soils.

Mean total Hg concentrations in potato samples in CK, LW_Hg_ and HG_Hg_ treatment groups were 0.54, 1.92 and 3.42 μg kg^−1^, respectably (Fig. 1). The highest (7.05 μg kg^−1^) and lowest (0.12 μg kg^−1^) total Hg concentrations were recorded in the HG_Hg_ and LW_Hg_ treatments in Shanxi and Shaanxi soil, respectively. In general, total Hg concentrations did not record a wide variation among the different potato samples (Fig. 1). A two-way ANOVA test was undertaken to further assess the effect and interaction of soil types and exposure dose on Hg concentration in the edible part of potatoes (Table 3). Results from this analysis indicated that there were significant associations and interactions between soil type, exposure dose and Hg contents in potatoes (p < 0.001). However, with reference to the limit of 10 μg kg^−1^ of Hg established by the national food safety standards in vegetables (GB 2762-2012)^25^, it can be considered that potatoes grown in slightly Hg-contaminated soils are safe for human consumption.

**Table 3:** A two-way ANOVA of the effects of soil type (S) and treatment (T) on potato edible part Hg content and yield.

Factors	DF	Potato Hg content			Yield		
		SS	F	P	SS	F	P
S	17	2.5	3.0	***	550843.8	22.0	***
T	2	33.3	346.1	***	853.3	0.3	0.7
S×T	34	83.6	51.1	***	1268012.0	2.53	***
model	53	426.1	166.9	***	1507752.6	19.3	***
error	108	5.2			159138.7		

***p < 0.001

### Bioconcentration of Hg

**Figure 2:**
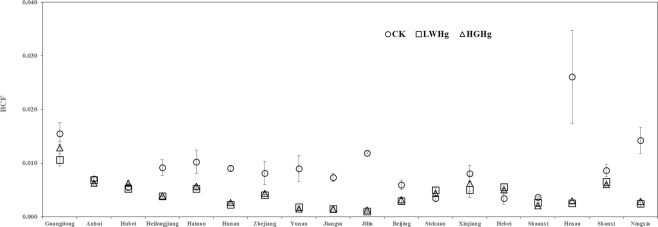
Bioconcentration factor (BCF) of Hg from soil to edible portion of potatoes in each Hg treatment.

Bioconcentration factors (BCFs) of Hg concentrations in edible parts of potatoes grown in the three treatment groups are shown in Fig. 2. Results indicate that all BCFs were below 0.04, suggesting that potato is a low accumulation/concentration crop. Based on average BCF values of Hg under different contaminated levels, samples in the CK treatment could accumulate Hg in the edible parts of potato at higher concentrations compared to the other two treatment groups (Table 4). Average and standard deviation results of BCFs in the three Hg treatment groups among different acid-alkaline soils (Table 4) indicated that average BCF values in contaminated treatment (LW_Hg_ and HG_Hg_) groups cascaded from alkaline soils → acid soils → neutral soil. Here, BCF values in alkaline soil were significantly higher than those recorded in the other two soil types (p < 0.05), indicating that a higher concentration of Hg accumulated in potatoes grown in contaminated alkaline soils.

**Table 4:** Bioconcentration factor (BCF) of Hg from soil to edible portion of potato in different soils (pH < 6.5, 6.5 < pH < 7.5, pH > 7.5) with three different Hg concentrations.

pH	CK	LW_Hg_	HG_Hg_
<6.5	0.0096 ± 0.003 aA^*^	0.0024 ± 0.001bB	0.0025 ± 0.001bB
6.5-7.5	0.0056 ± 0.003 aA	0.0015 ± 0.001bB	0.0013 ± 0.001bB
>7.5	0.0063 ± 0.005 aA	0.0038 ± 0.002aB	0.0042 ± 0.002aAB

*Mean ± SD, different small letters within the same column and different capital letters within the same row for each treatment indicate a significant difference at p < 0.05 by Student’s multiple range tests.

### Potato tuber yield

Potatoes grown in soil with a pH higher than 7.5 recorded the highest average yields (251.1, 269.9 and 255.9 g pot^−1^ in the CK, LW_Hg_ and HG_Hg_ treatment groups, respectively) compared with lower soil pH groups (Table 5). It was evident that potatoes grown in soil collected from Anhui and Hainan regions did not display any visual symptoms of stress, however they were noted to be generally smaller. In addition, edible biomass in LW_Hg_ and HG_Hg_ treatment groups did not significantly change compared to potatoes grown in the CK treatment group (Table 5). Results gained from two-way ANOVA test indicated that there were no significant differences between Hg exposure dose and potato yield (Table 3).

**Table 5:** Effect of treatments on potato yields in pots with different soils (pH < 6.5, 6.5 < pH < 7.5, pH > 7.5) with three different Hg concentrations at the end of the experiment.

pH	soil location	CK	LW_Hg_	HG_Hg_
<6.5	Guangdong	213.1 ± 18.1	194.9 ± 14.0	217.4 ± 33.5
	Anhui	50.4 ± 5.1	77.8 ± 13.7	52.6 ± 5.2
	Hubei	123.4 ± 9.0	128.3 ± 24.7	113.6 ± 17.4
	Heilongjiang	186.8 ± 17.4	202.7 ± 21.2	322.1 ± 43.8
	Hainan	47.4 ± 6.4	26.0 ± 4.4	26.2 ± 3.6
	average	124.2 ± 71.6bA	125.9 ± 72.4bA	146.4 ± 116.8bA
6.5-7.5	Hunan	280.3 ± 28.7	351.7 ± 26.0	308.7 ± 30.1
	Zhejiang	166.0 ± 10.1	193.6 ± 33.0	189.9 ± 25.8
	Yunan	166.3 ± 20.9	201.9 ± 11.9	240.1 ± 40.3
	Jiangsu	302.0 ± 15.2	273.2 ± 22.5	225.6 ± 76.0
	average	228.7 ± 69.0 aA	255.1 ± 74.3 aA	241.1 ± 66.8 aA
>7.5	Jilin	413.7 ± 18.3	367.6 ± 52.6	368.1 ± 22.5
	Beijing	186.3 ± 5.7	294.8 ± 12.6	294.5 ± 40.0
	Sichuan	414.6 ± 14.6	381.7 ± 77.2	337.9 ± 16.3
	Xinjiang	277.1 ± 61.7	260.9 ± 12.3	261.8 ± 70.3
	Hebei	184.0 ± 12.5	281.0 ± 43.8	294.0 ± 10.7
	Shaanxi	176.0 ± 12.1	178.5 ± 19.0	150.4 ± 30.2
	Henan	259.7 ± 76.9	249.2 ± 10.3	259.3 ± 12.2
	Shanxi	236.3 ± 29.7	245.7 ± 10.1	235.4 ± 39.4
	Ningxia	112.4 ± 17.8	170.0 ± 11.6	102.0 ± 18.8
	average	251.1 ± 104.0 aA	269.9 ± 78.8 aA	255.9 ± 88.8 aA

*Mean ± SD, different small letters within the same column and different capital letters within the same row for each treatment indicate a significant difference at p < 0.05 by Student’s multiple range tests.

## Discussion

Analysis using two-way ANOVA indicated that Hg concentrations in potato tubers was significantly affected by soil type, soil Hg concentration and their interactions (Table 3). These findings confirm that soil type and soil Hg contamination level can regulate Hg uptake by potatoes^26,27^. Results in the two Hg contamination groups recorded alkaline soils to have the lowest average available Hg/total Hg ratios, regardless of sampling before or after potato planting, and the highest average ratios were recorded in acidic soils. These findings were in line with our expectations. Previous studies have also reported that soil acidification is the most important factor for a higher metal fraction in soils and for metal uptake by plants^28,29^. The correlation between soil parameters and Hg concentrations in edible parts of various crop species were examined by Hu *et al*.^30^ using stepwise multiple linear regression analysis; results indicated that soil pH and OM are the two most important parameters. Additionally, Ding *et al*.^13^, using the path analysis method, recorded that pH and free Al oxide (Al_OX_) are the most essential soil parameters correlated with Hg concentrations in carrots.

Moreover, our results indicated that Hg concentrations in potatoes displayed a strong positive correlation with total soil Hg concentrations, similar to previous findings^31,32^. However, it has been widely reported that plants mainly absorb and utilize available Hg, and it can act as a crucial indicator for the adsorption capability of heavy metals in soils^33^. In our experiments, no significant correlation was recorded in the available Hg concentration between soil and potato tubers. That is to say, recorded levels of EDTA-extractable soil Hg concentrations may not able to indicate the amount of soil metals plants uptake. This finding is probably due to several reasons: (i) When available Hg is reduced by crop uptake, potentially available forms may supplement this uptake to ensure equilibrium is achieved^34^. (ii) In addition to residual Hg, the potential available state can be directly absorbed by plants under certain conditions^35^, mainly being attributed to soil properties, soil ion effects and plant species. (iii) Due to the high level of starch present in potato tubers, this root vegetable differs from other root vegetables, resulting is this underlying phenomenon. It can therefore be considered that Hg bioavailability in a soil is not only associated with basic soil properties, it is also related to the mechanisms of migration and transformation of Hg in plants.

Zhao *et al*.^4^ suggested that a soil sample can be considered as slightlycontaminated when its metal concentration is 1–3 times higher than benchmark values. And in our result, slight Hg contamination did not affect potato yield. This finding may be attributed to the detoxification mechanism of soil and plants. Specially, soil microbes can become more resistant to higher Hg concentrations^36^, and the most significant bacterial Hg resistance mechanism is through the reduction of Hg^2+^ to volatile Hg0 catalyzed by the merA gene^37^. In addition, Hg-tolerance mechanisms of potatoes may act by eliminating the detrimental effects of Hg^38^, such as preventing Hg^2+^ from interfering with cell metabolic pathways via metal immobilization in the cell walls^39^, or metal chelation by organic acids and specific peptides^40^. Interestingly, among the three treatment groups, average potato yield recorded from plants grown in acidic soil were significantly lower than yields from the other two soils. Potato yield percentages were relatively similar to those reported by Luo^41^ from plants grown in acidic soils in Hunan, China. Furthermore, Pan *et al*.^42^ recorded that reduced pH values and increased exchangeable Al^3+^concentrations can inhibit plant growth and limit nutrient uptake. These observations suggest that acidic soil is not suitable for the growth of potatoes.

## Materials and methods

### Soil collection

Eighteen soil samples, representative of 13 different soil types (having different chemical and physical characteristics) were collected across mainland China (Table S1). Soil samples were collected from the upper soil layer (0-20 cm) from typical farmland ecosystems. Soil samples were thoroughly mixed, transported back to the laboratory and air-dried at room temperature. After drying, soil samples were passed through a 2-mm sieve before being used as the planting medium for potato plants. The chemical and physical characteristics of the soils were determined using conventional analytical methods.

### Experimental design

Experiments in our study included two variables (mercury treatment and soil type) and three replicates; all experiments were conducted in a greenhouse in Tianjin, China (39°5′49″N, 117°8′47″E). According to the Chinese environmental quality standard for soils released by the Ministry of Environmental Protection in 1995(GB15618-1995), Class II values (depending on soil pH and land use) can be applied to protect human health and agricultural production through the food chain (Table S2). Based on this information, we selected three Hg concentrations for the 18 soils: CK, a control sample that was not contaminated; low dosage LW_Hg_ (1 time environmental quality standard, grade II for soil mercury); and high dosage HG_Hg_ (2 times environmental quality standard, grade II for soil mercury). Soils were artificially contaminated with Hg (dissolved mercury appeared as Hg(NO_3_)_2_), and then aged for 90 days at room temperature. Potato seeds were sown on March 17, 2018, and harvested on June 24, 2018.

### Potato planting and management

Potato tubers (about 20 g per tuber) of Cultivars Zihuabai from China were used in this experiment. Four days before sowing, experimental soil placed in pots were adjusted using locally available and adapted fertilizers, resulting in: 3 g N pot^−1^, 2 g P pot^−1^ and 2 g K pot^−1^. Planting depth was 4 ∼ 6 cm. All pots were watered once a week in the seedling and tuber expansion periods, every ten days in the early florescence period, and every 15 days in the maturity period.

### Soil sampling and determination

All soils were sampled before potato tubers were planted on March 10 and after harvest on June 30. Total and available Hg concentrations in the soil samples were determined using the following methods:

1. Determination of total Hg content: Air-dried soil samples were crushed and passed through a 100-mesh sieve. Approximately 0.5 g of the soil was accurately weighed and transferred into a 50 ml colorimetric tube. 10 ml of aqua regia was the added to the tube and thoroughly shaken after stirring. The aqua regia solution was then boiled for 2 hours to ensure sample dissolution; during this process samples were intermittently shaken. After cooling, 10 ml of potassium citrate preservation solution was added to the samples before they were diluted to 50 ml. Finally, supernatant was collected and Hg concentration was determined using an atomic fluorescence spectrometer (AFS-3100, Beijing Haiguang Instrument Co., Ltd.).
2. Determination of valid Hg concentration: Air-dried soil samples were crushed and passed through a 100-mesh sieve. Approximately 5 g of soil was then accurately weighed and transferred into a 100 mL flask. 50 ml of 0.05 mol/l EDTA extractant was then added to the samples. Samples were vigorously shaken for 1 hour at 25 °C before being filtered. Valid Hg concentrations were then determined by analyzing the filtrate using an atomic fluorescence spectrophotometer.

### Vegetable sampling and determination

On June 24 (99 days after transplanting), potatoes were harvested. Plant samples were initially washed with tap water before being rinsed with deionized water. Surface water was removed using absorbent paper. Biomass of the edible part was recorded (fresh weight) using an electronic balance and total Hg concentration in the plant samples was determined.

Total Hg concentrations were determined using potato samples that were homogenized using a masher. 1.0 g of sample was weighed and transferred into 50 ml colorimetric tubes with a plug. After acid (HNO_3_:HClO_4_ = 4:1, v/v) was added to the samples, the tubes were stored overnight. On the next day, samples were heated in a boiling water bath for 2 hours; samples were intermittently shaken during this period. Following complete dissolution, sample volume was made up to 50 ml using a potassium dichromate solution. After being shaken, the supernatant was collected and Hg concentration was determined using an atomic fluorescence spectrophotometer.

### Statistical analysis

All statistical analyses were conducted using JMP 9.0. Statistical differences among treatment groups were compared using one-way analysis of variance (ANOVA). Correlations between soil total/available Hg concentrations and potato edible Hg concentrations were evaluated using Pearson’s correlation coefficient. Statistical differences among soil type, soil Hg treatment, potato Hg concentration and potato yield were analyzed using two-way ANOVA.

## Conclusions

Results from our study indicate that Hg concentration in the edible parts of potatoes were under acceptable limits (<10 μg kg^−1^) and the BCF values for potatoes were below 0.04. These results suggest that potatoes grown in Hg contaminated soil posed no significant health risks. Although potato growth was recorded to be affected by soil pH, our results indicated that potatoes grew normally in soils which were slightly contaminated by Hg. Moreover, findings from our study indicate that the effectiveness of soil Hg may not be a good predictor for Hg uptake by potatoes. Our results provide additional information for improving current understanding of the accumulation behavior of Hg in potatoes, providing important information for the evaluation of food safety and potatoes in China.

## Supplementary information

Supplementary Information.
