# Supplementary material for: Potato (Solanum tuberosum L.) can be grown safety on human consumption in slight Hg-contaminated soils across China mainland
Source: Sci Rep. 2020 May 20;10:8351. doi: 10.1038/s41598-020-65430-1 (PMC7239881; doi:10.1038/s41598-020-65430-1)
Supplement: Supplementary file 1 — Supplementary Information. [file 41598_2020_65430_MOESM1_ESM.docx]

Table S1 Physical and chemical properties of the tested soil

| Area | Soil Type | pH | OM | CEC | Al_Ox_ | Fe_Ox_ | Clay | Background Hg |
| --- | --- | --- | --- | --- | --- | --- | --- | --- |
|  |  |  | g kg^-1^ | cmol kg^-1^ | g kg^-1^ | g kg^-1^ | <0.002mm | mg kg^-1^ |
| Guangdong | Red soil | 4.91 | 46.09 | 18.00 | 1.37 | 20.52 | 39.94 | 0.04 |
| Anhui | Yellow-brown earths | 5.29 | 21.88 | 14.41 | 2.86 | 23.93 | 29.64 | 0.14 |
| Hubei | Paddy soils | 5.61 | 29.48 | 9.81 | 1.23 | 11.28 | 24.44 | 0.11 |
| Heilongjiang | Black soils | 5.78 | 54.71 | 33.41 | 1.67 | 7.42 | 32.80 | 0.05 |
| Hainan | Humid-thermo ferralitic | 6.07 | 14.37 | 10.583 | 4.97 | 79.08 | 36.44 | 0.07 |
| Hunan | Paddy soils | 6.60 | 27.61 | 13.90 | 2.61 | 23.34 | 33.74 | 0.12 |
| Zhejiang | Paddy soils | 6.82 | 33.43 | 20.42 | 0.69 | 8.07 | 26.24 | 0.07 |
| Yunnan | Yellow-red earths | 6.93 | 26.52 | 16.13 | 1.03 | 15.18 | 24.74 | 0.07 |
| Suzhou | Paddy soils | 7.44 | 22.56 | 18.43 | 1.05 | 13.06 | 21.76 | 0.04 |
| Jilin | Black soils | 7.92 | 33.30 | 28.26 | 1.26 | 7.04 | 36.18 | 0.06 |
| Beijing | Black soils | 8.09 | 11.60 | 19.88 | 0.56 | 5.86 | 20.61 | 0.07 |
| Sichuan | Purplish soils | 8.09 | 15.13 | 10.40 | 0.57 | 7.05 | 15.58 | 0.04 |
| Xinjiang | Gray desery soils | 8.17 | 14.22 | 10.59 | 0.58 | 4.03 | 18.98 | 0.04 |
| Hebei | Fluvo-aquic soils | 8.21 | 7.90 | 9.50 | 0.56 | 5.97 | 14.35 | 0.04 |
| Shaanxi | Loessial soils | 8.25 | 14.60 | 10.54 | 0.52 | 6.58 | 15.12 | 0.04 |
| Henan | Fluvo-aquic soils | 8.35 | 12.18 | 10.10 | 0.52 | 7.38 | 11.09 | 0.04 |
| Shanxi | Castanozems | 8.39 | 21.63 | 17.28 | 0.61 | 7.27 | 16.69 | 0.07 |
| Ningxia | Calcic soils | 8.89 | 3.31 | 4.87 | 0.31 | 4.11 | 12.52 | 0.04 |

Table S2 The Grade Two Standard in the Soil Environmental Quality Standards of China for Hg (GB15618-1995) and the amounts of Hg added as Hg(NO_3_)_2_ (mg kg^–1^)

| pH | <6.5 | 6.5–7.5 | >7.5 |
| --- | --- | --- | --- |
| Grade Two Standard | 0.3 | 0.5 | 1.0 |
| Low Hg | 0.3 | 0.5 | 1.0 |
| High Hg | 0.6 | 1.0 | 2.0 |
